# I Say IOS You Say AOS: Comparative Bias in Respiratory Impedance Measurements

**DOI:** 10.1007/s00408-019-00247-y

**Published:** 2019-07-04

**Authors:** Chris RuiWen Kuo, Sunny Jabbal, Brian Lipworth

**Affiliations:** Scottish Centre for Respiratory Research, Ninewells Hospital and Medical School, University of Dundee, Dundee, Scotland DD1 9SY UK

**Keywords:** Impulse oscillometry, Airwave oscillometry, Asthma, COPD, Spirometry, Asthma control questionnaire

## Abstract

**Background:**

The forced oscillation technique (FOT) measures respiratory impedance during normal tidal breathing and requires minimal patient cooperation.

**Objective:**

To compare IOS and AOS devices in patients with asthma and COPD.

**Methods:**

We compared two different FOT devices, namely impulse oscillometry using a loudspeaker (IOS: Jaeger Masterscreen) and airwave oscillometry using a vibrating mesh (AOS: Thorasys Tremoflo) for pre- and post-bronchodilator measurements in 84 patients with asthma and COPD.

**Results:**

The overall pattern of measurement bias was for higher resistance with IOS and higher reactance with AOS, this being the case in asthma and COPD separately. There were small but significantly higher values using IOS for resistance at 5 Hz (R5) and 20(19) Hz (R20(19)). In converse, values for reactance at 5 Hz (X5), reactance area (AX) and resonant frequency (Fres) were significantly higher using AOS but to a much larger extent. The difference in AX between devices was more pronounced in COPD than in asthma. Salbutamol reversibility as % change was greater in asthma than COPD patients with AX but not FEV1.

**Conclusion:**

Our study showed evidence of better agreement for resistance than reactance when comparing IOS and AOS, perhaps inferring that AOS may be more sensitive at measuring reactance in patients with airflow obstruction.

**Electronic supplementary material:**

The online version of this article (10.1007/s00408-019-00247-y) contains supplementary material, which is available to authorised users.

## Introduction

Current guidelines for asthma and COPD advocate the use of spirometry to quantify the degree of airflow obstruction [1, 2]. Spirometry involves performing an artificial forced expiratory manoeuvre from total lung capacity to residual volume. As such spirometry induces volume dependent small airway closure. Patients often find it difficult to perform an adequate procedure forcibly breathing out all the way to residual volume, especially in those who are coughing or breathless. Hence, an alternative easier way of assessing lung function is required for patients with asthma and COPD.

One such method is the so-called forced oscillation technique (FOT) which was originally described by Dubois [3] using a single frequency and subsequently refined with multiple frequencies by Michaelson [4]. FOT involves measuring the pressure/flow (kPa/l.s) relationship while forced oscillations of sound waves are imposed upon normal tidal breathing to determine respiratory impedance [5]. Different FOT methods have been developed with two commonly used commercial devices being impulse oscillometry using a loudspeaker source (IOS, Jaeger Masterscreen, Carefusion Hoechberg, Germany) and airwave oscillometry using a vibrating mesh (AOS, Tremoflo, Thorasys, Montreal).

The application of IOS to asthma and COPD has been previously described in detail elsewhere [6, 7]. In brief the nature of the sound waveform determines the frequencies at which the respiratory impedance is measured within the bronchial tree. Both methods can be crudely thought of as being akin to bronchial sonar using bidirectional harmonic sound waves between 5 and 35 Hz. The measured respiratory impedance in turn comprises components of in-phase resistance (R) and out-of-phase reactance (X). For the resistance component of impedance, the measurement at 5 Hz (R5) is thought to reflect the total lung resistance, while at 20 Hz (R20) reflects central lung resistance. The difference between resistance at 5 Hz and 20 Hz (R5–R20) represents the frequency-dependent heterogeneity and in essence refers to peripheral lung resistance (i.e. total minus central)[8], which in turn is related to long-term asthma control [9].

The reactance component reflects the balance of inertial and elastic properties of the distensible lung tissue and airways. This is normally measured at a low frequency of 5 Hz (X5) which is denoted as a negative value, along with the area under the reactance curve (AX) between 5 Hz and the resonant frequency (Fres) where the reactance curve crosses the zero line. AX essentially represents where elastance surpasses inertance at lower frequencies, with higher values (denoted as positive numbers) reflecting reduced lung compliance and hence stiffer lungs. Pointedly AX has been shown to be related to exacerbations of COPD as well as to asthma control [10–13]. In asthma, changes in AX and R5 are concordant in relation to increasing disease severity, while AX is more affected than R5 in relation to disease severity in COPD [14–16]. R5–R20 and AX in particular are thought to reflect changes in the more distal small airways, the so-called quiet zone of the lung [17].

The primary objective of the present study was to compare head to head the IOS and AOS devices in patients with asthma and COPD.

## Patients and Methods

Retrospectively we evaluated IOS and AOS readings of a cohort of 84 adult patients who voluntarily attended our centre for potential screening into clinical trials. Patients included into this study had established diagnosis of either asthma or COPD and were all on prescribed inhaler therapy at the time of visit. During the visit, spirometry, IOS and AOS were performed and asthma patients were asked to complete the six point Asthma Control Questionnaire (ACQ) [18, 19]. Spirometry (Micromedical, Chatham, United Kingdom) was performed in triplicate according to European Respiratory Society guidelines, always done after IOS and AOS measurements. IOS and AOS measurements were done in random order and performed in triplicate according to guidelines [5] using Jaeger Masterscreen IOS system and Thorasys TremoFlo AOS system. Accuracy of resistance measurements was confirmed on each day with a 3L calibration syringe and a standard 0.2 kPa/l.s resistance mesh.

For both IOS and AOS, participants were seated wearing a nose clip, with both hands supporting their cheeks with normal tidal breathing. IOS, AOS and spirometry were measured before and after bronchodilator as inhaled salbutamol 400 µg.

Following 40 s of tidal breathing for IOS and 20 s for AOS, measurements of R5, R20 (R19 for AOS), Fres, AX and X5 were generated. Each test was inspected for artefacts and a minimum of three measurements were obtained. Readings with coherence values of ≥ 0.7 at 5 Hz were considered acceptable and a between test coefficient of variation of Zrs < 15%. In cases where Fres > 35 Hz, we considered that the associated AX values could not be properly calculated and hence such measurements were excluded from the dataset, although those for X5 remained valid. As the default setting for the Tremoflo device measures resistance at 19 Hz (R19), we compared R20 for IOS versus R19 for AOS in all subjects, and in a subgroup of *n* = 58 subjects, after a subsequent software update, we were able to also ascertain R20 for AOS and hence directly compare with R20 for IOS.

## Statistical Analysis

The data were initially inspected for normal distribution. Bland–Altman analysis was applied to identify the comparative bias between the measurements of two devices. The Bland–Altman data for differences were then analysed with linear regression models to evaluate the degree of bias between IOS and AOS measurements. Comparisons of mean differences between devices were performed using pairwise Student’s *t* tests. Linear regression models were also separately applied to IOS and AOS measurements to assess the model fit. In addition, linear regression was applied to examine the relationship between AX versus FEV_1_% predicted and versus ACQ, the latter only in asthma patients. *p* Values are quoted as < 0.05, < 0.01, < 0.001.

## Results

Demographic data on the combined and separate asthma and COPD groups are shown in Table 1. As expected, this showed that patients with COPD had lower FEV_1_ (*p* < 0.001), FEF25-75 (*p* < 0.001), FEV_1_/FVC ratio (*p* < 0.001); higher R5–R20(19) (*p* < 0.05), AX (*p* < 0.001) and Fres (*p* < 0.05); lower X5 (*p* < 0.05) values compared to those with asthma. Patients with COPD were heavier smokers, while asthma patients were more atopic. Mean coherence values for impedance at 5 Hz and 20 Hz were 0.80 and 0.94 respectively for IOS and 0.89 and 0.95 for AOS.

**Table 1:** Demographic data

	Overall *N* = 84		Asthma *N* = 59		COPD *N* = 25	
Age	54		50		65	
Gender F/M	46/38		34/25		12/13	
BMI	29		30		28	
Smoking status n/e/c	39/36/9		39/19/1		0/8/17	
Smoking pack year history	14		4		40	
SPT	–		2		–	
ICS (µg)	770		770		810	
LABA	56		39		17	
LAMA	26		8		18	
LTRA	20		20		0	
THEO	2		2		0	
OAH	25		24		1	
ACQ	–		1.60		–	
FEV_1_ (% predicted)	81		88		64	
FEF_25–75_ (% predicted)	45		54		24	
FVC (% predicted)	101		102		99	
FEV1/FVC ratio	0.66		0.72		0.52	

	IOS	AOS	IOS	AOS	IOS	AOS
R5 (kPa/l.s)	0.52	0.50	0.50	0.49	0.58	0.53
R20(19) (kPa/l.s)	0.37	0.36	0.37	0.36	0.38	0.36
R5–R20(19) (kPa/l.s)	0.15	0.15	0.13	0.13	0.20	0.17
AX (kPa/l)	1.42	2.47	1.10	1.96	2.13	3.59
X5 (kPa/l.s)	− 0.23	− 0.29	− 0.20	− 0.24	− 0.29	− 0.40
Fres (Hz)	18.63	23.62	17.38	21.96	21.43	27.35

Smoking status n/e/c = non/ex/current smoker, ICS as beclomethasone µg equivalent dose

*SPT* skin prick test, *LABA* long acting *β*^2^ agonist, *LAMA* long acting muscarinic antagonist, *LTRA* leukotriene receptor antagonist, *THEO* theophylline, *OAH* oral antihistamine. The conversion factor from kPa to cmH_2_O is × 10.2

**Fig. 1:**
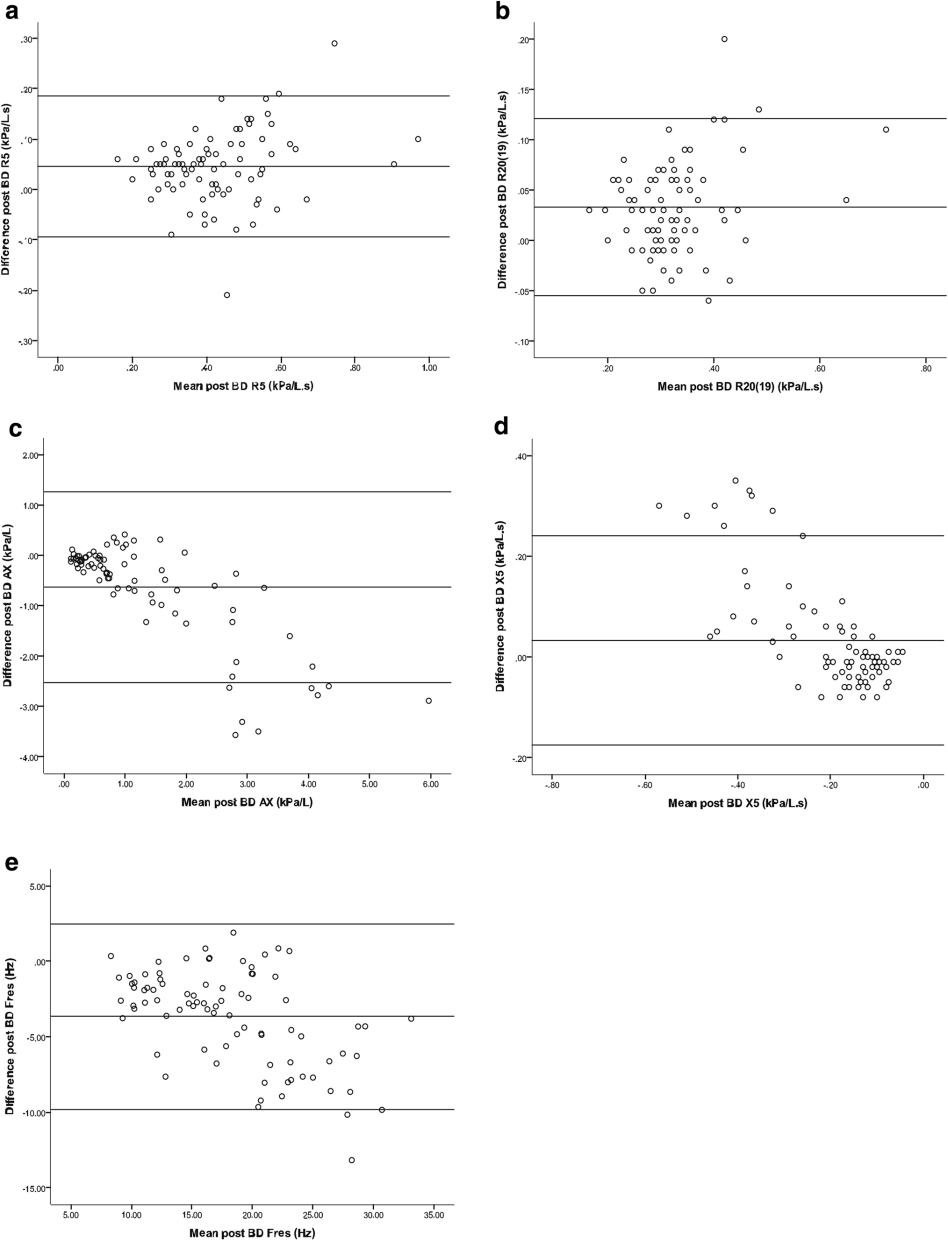
Bland–Altman plots in all patients (i.e. asthma and COPD) showing post-bronchodilator values of **a** R5; **b** R20 (19); **c** AX; **d** X5 and **e** Fres. The conversion factor from kPa to cmH_2_O is × 10.2

**Fig. 2:**
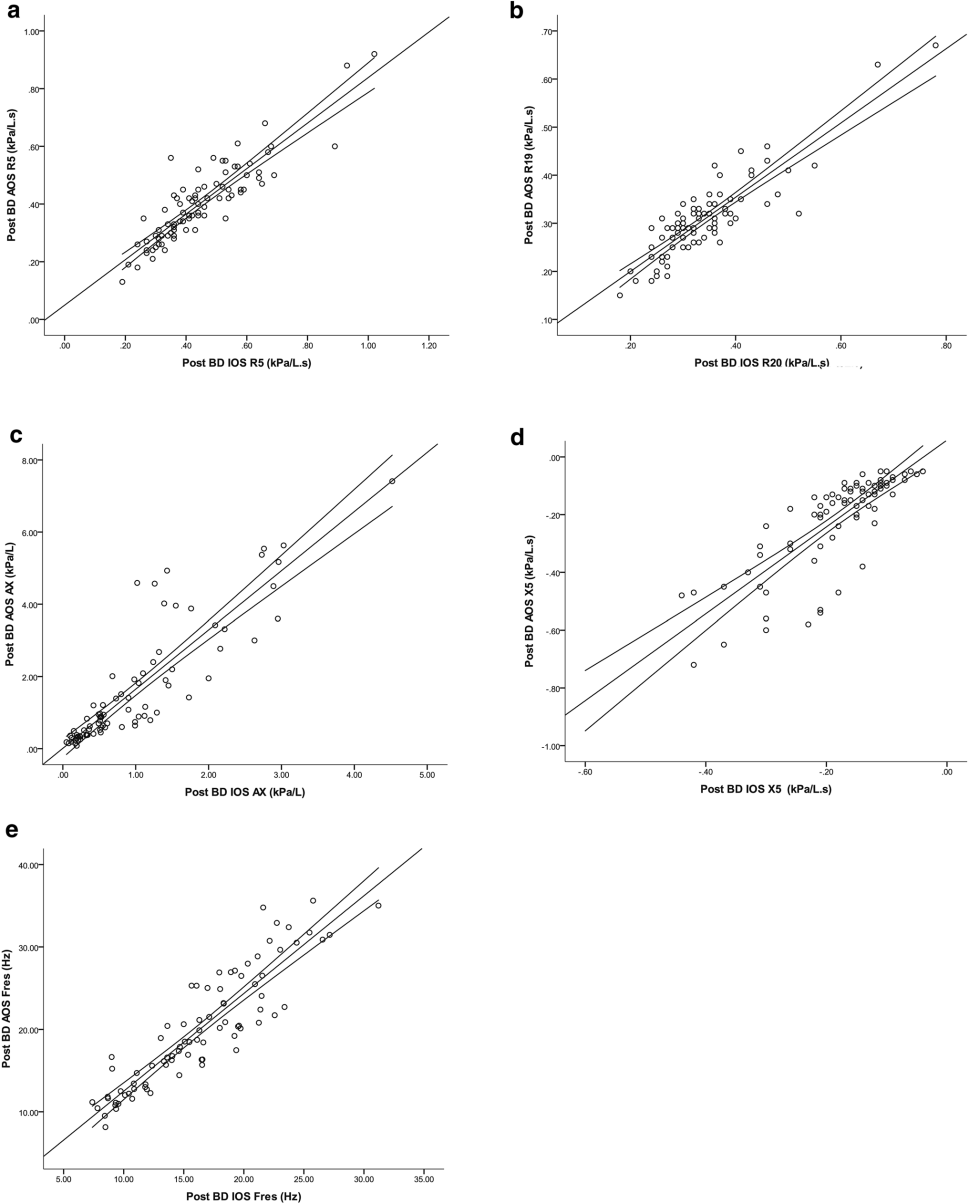
Scatter plot in all patients (i.e. asthma and COPD) of **a** R5; **b** R20(19); **c** AX; **d** X5 and **e** Fres showing linear regression line of best fit and 95% CI for IOS versus AOS. *R*^2^ and intra-class correlation coefficient (ICC) values were R5 *R*^2^ = 0.79, ICC = 0.94; R20(19) *R*^2^ = 0.77, ICC = 0.93; AX *R*^2^ = 0.78, ICC = 0.85; X5 *R*^2^ = 0.65, ICC = 0.8; Fres *R*^2^ = 0.82, ICC = 0.93 (*p* < 0.001 for all regression models). The conversion factor from kPa to cmH_2_O is × 10.2

Bland–Altman plots for resistance revealed good agreement between IOS and AOS with R5 and R20 (Fig. 1). Regression analysis of the Bland–Altman differences between devices showed a weakly significant poor model fit for R5 (*R*^2^ = 0.06, *p* < 0.05) and for R20(19) (*R*^2^ = 0.07, *p* < 0.05). The degree of comparative bias between devices was small showing relatively higher values with IOS, and such differences were significant for post-bronchodilator R5, R20(19) and R5–R20(19) (Table 2). Regression analysis of IOS versus AOS values for R5 and R20(19) showed highly significant model fits along with narrow confidence intervals, *R*^2^ and ICC values exceeding 0.7 and 0.9, respectively (Table 2 and Fig. 2). Similar results between IOS versus AOS were obtained for a subset of *n* = 58 patients who had R20 measured with AOS: *R*^2^ = 0.83, *p* < 0.001, ICC = 0.95, mean difference = 0.034 kPa/l.s (95% CI 0.023, 0.045; *p* < 0.001).

**Table 2:** Comparison of IOS and AOS measurements

IOS versus AOS	R^2^		ICC		Mean diff (CI)	
	Pre BD	Post BD	Pre BD	Post BD	Pre BD	Post BD
Overall						
R5 (kPa/l.s)	0.74^†^	0.79^†^	0.92	0.94	0.018 (− 0.002, 0.038)	0.046 (0.030, 0.061)**
R20(19) (kPa/l.s)	0.74^†^	0.77^†^	0.92	0.93	0.016 (0.005, 0.027)*	0.033 (0.023, 0.043)**
R5–R20(19) (kPa/l.s)	0.68^†^	0.66^†^	0.90	0.90	0.003 (− 0.012, 0.017)	0.012 (0.001, 0.023)*
AX (kPa/l)	0.70^†^	0.78^†^	0.84	0.85	− 1.042 (− 1.323, − 0.762)**	− 0.639 (− 0.852, − 0.426)**
X5 (kPa/l.s)	0.63^†^	0.65^†^	0.82	0.80	0.061 (0.034, 0.089)**	0.033 (0.010, 0.056)*
Fres (Hz)	0.81^†^	0.82^†^	0.92	0.93	− 4.990 (− 5.773, − 4.210)**	− 3.666 (− 4.353, − 2.980)**
Asthma						
R5 (kPa/l.s)	0.72^†^	0.73^†^	0.92	0.92	0.006 (− 0.018, 0.029)	0.035 (0.016, 0.053)**
R20(19) (kPa/l.s)	0.73^†^	0.75^†^	0.92	0.93	0.015 (0.004, 0.027)*	0.025 (0.015, 0.036)**
R5–R20(19) (kPa/l.s)	0.69^†^	0.52^†^	0.91	0.84	− 0.009 (− 0.025, 0.008)	0.008 (− 0.005, 0.022)
AX (kPa/l)	0.59^†^	0.57^†^	0.78	0.71	− 0.857 (− 1.195, − 0.518)**	− 0.443 (− 0.668, − 0.218)**
X5 (kPa/l.s)	0.47^†^	0.49^†^	0.77	0.70	0.040 (0.010, 0.070)*	0.011 (− 0.013, 0.036)
Fres (Hz)	0.77^†^	0.79^†^	0.92	0.91	− 4.578 (− 5.560, − 3.596)**	− 3.405 (− 4.217, − 2.593)**
COPD						
R5 (kPa/l.s)	0.79^†^	0.86^†^	0.94	0.96	0.047 (0.013, 0.082)*	0.071 (0.044, 0.098)**
R20(19) (kPa/l.s)	0.75^†^	0.82^†^	0.92	0.94	0.018 (− 0.006, 0.042)	0.051 (0.029, 0.072)**
R5–R20(19) (kPa/l.s)	0.66^†^	0.76^†^	0.89	0.93	0.029 (0.001, 0.057)*	0.021 (0.004, 0.039)*
AX (kPa/l)	0.78^†^	0.84^†^	0.87	0.88	− 1.453 (− 1.949, − 0.956)**	− 1.105 (− 1.559, − 0.651)**
X5 (kPa/l.s)	0.76^†^	0.66^†^	0.85	0.83	0.111 (0.054, 0.168)*	0.083 (0.035, 0.131)*
Fres (Hz)	0.83^†^	0.78^†^	0.92	0.92	− 5.922 (− 7.195, − 4.649)**	− 4.272 (− 5.614, − 2.930)**

*BD* bronchodilator (salbutamol 400 µg), *ICC* intra-class correlation coefficient. *R5* resistance at 5 Hz, *R20* resistance at 20 Hz, *AX* area under the reactance curve, *X5* reactance at 5 Hz, *Fres* resonant frequency

^†^*p* < 0.001 for regression model fit

*p < 0.05; **p < 0.001 for mean difference IOS–AOS

Bland–Altman plots for reactance showed poor agreement between IOS and AOS for AX and X5 along with a similar trend for Fres (Fig. 1). Regression analysis of the Bland–Altman differences between devices showed a highly significant model fit for AX: (*R*^2^ = 0.66, *p* < 0.001), X5 (*R*^2^ = 0.56, *p* < 0.001) and Fres (*R*^2^ = 0.29, *p* < 0.001). The degree of comparative bias between devices was large showing relatively higher values with AOS for AX and Fres and lower values for X5, with highly significant differences in both pre- and post-bronchodilator values (Table 2). As expected, mean differences between devices were smaller for post- compared to pre-bronchodilator values of AX, X5 and Fres.

Regression analysis of IOS versus AOS showed a highly significant model fit with wider confidence intervals at higher AX and Fres values and at lower X5 values. *R*^2^ values ranged between 0.6 and 0.8 and ICC values between 0.8 and 0.9 (Table 2, Fig. 2).

Looking at asthma and COPD patients separately, the same trend was seen in terms of comparative bias between device for post-bronchodilator values, with relatively higher R5 (*p* < 0.05), R20(19) (*p* < 0.05) for IOS, along with higher AX (*p* < 0.05), and lower X5 (*p* < 0.01) for AOS (Table 2). The magnitude of differences between devices for resistance, reactance and Fres were all greater in patients with COPD than in asthma.

The % reversibility was similar for FEV_1_ comparing asthma (8.14%) and COPD (8.40%), while for AX the degree of reversibility was more pronounced in asthma than COPD with AOS: 40% versus 24% (*p* < 0.05) and IOS: 32% versus 19% (*p* > 0.05).

Regression analysis for post-bronchodilator AX and X5 in relation to FEV_1_% predicted in the overall population showed a highly significant model fit of moderate effect size for both devices (Fig. E1). However, post-bronchodilator AX exhibited a weaker model fit in relation to ACQ for either IOS (*R*^2^ = 0.16, *p* < 0.01) or AOS (*R*^2^ = 0.04, *p* = 0.143). ROC analysis for post-bronchodilator R5 showed a significant AUC for IOS but not AOS in relation to ACQ value of 0.75: AUC = 0.75 (95% CI 0.61, 0.89), *p* < 0.01, sensitivity = 0.79, specificity = 0.6, for a R5 cut point of 0.36 kPa/l.s. ROC analysis for post-bronchodilator AX showed a significant AUC for IOS but not AOS in relation to ACQ value of 1.0: AUC = 0.66 (95% CI 0.51, 0.81), *p* < 0.05, sensitivity = 0.62, specificity = 0.62, for a AX cut point of 0.52 kPa/l.

## Discussion

The main findings of the present study were a small degree of comparative bias between devices for resistance as R5 and R20(19) with relatively higher values being reported with IOS. Conversely, respective values for reactance (as X5 and AX) and Fres were relatively higher with AOS but to a much larger extent. For the combined population of asthma and COPD patients, there was good agreement from the Bland–Altman analysis between IOS and AOS for resistance measured at 5 Hz and 20(19)Hz with a small but significant bias in terms of higher post-bronchodilator values with IOS versus AOS at both frequencies. The mean differences in R5 and R5–R20(19) prior to salbutamol were not however statistically significant, presumably reflecting the wider variance associated with larger pre-bronchodilator resistance values.

In the combined population for reactance, there was a much higher degree of comparative bias between devices for AX and X5 values pre and post salbutamol, which was also mirrored by the difference in Fres. This was clearly evident from regression analysis of the Bland–Altman plots where there was a highly significant model fit for the differences. On inspection, the divergence in values between devices became more apparent at higher values of AX above 2 kPa/l. To put this degree of bias in context, the overall mean difference in pre-bronchodilator AX was 1.04 kPa/l amounting to a 73% higher value for AOS than IOS, while for post-bronchodilator AX the difference was 0.64 kPa/l, representing a 65% respective higher value. We believe the magnitude of such difference in AX is likely to be clinically relevant, although we duly acknowledge the lack of any defined minimal clinically important difference values.

The higher AX values seen with AOS than IOS at first glance might infer that AOS is more sensitive at detecting changes in low frequency reactance, in turn suggesting that the IOS is perhaps under reading compared to AOS. We believe that this is more likely to be the case than over reading with AOS per se. One possible explanation for the reactance bias is that the pulsed waveforms with IOS are based around a fundamental frequency of 5 Hz, with lower amplitudes occurring at higher frequencies. In contrast, AOS exhibits equivalent amplitudes across a range of non-harmonic prime frequencies and may therefore be less susceptible to distortion. Differences in factory calibration settings and breathing patterns might in part explain the small bias observed in resistance between devices but are unlikely to account for the much larger bias in reactance. One possible solution would be to have defined reference standards for both reactance and resistance in regard to device calibration.

One of the strengths of our study was the inclusion of both asthma and COPD patients which resulted in being able to compare the devices over a wide range of reactance and resistance values. On the other hand, a weakness is that we did not have a comparator group of healthy controls. Soares et al. [20] compared the same IOS and AOS devices including two age-matched control groups, one non-smokers and the other symptomatic current smokers. They observed the same overall trends of comparative bias as we did, amounting to mean differences in post-bronchodilator R5 and AX of 0.03 kPa/l.s and 0.16 kPa/l respectively in non-smokers, 0.06 kPa/l.s and 0.33 kPa/l respectively in smokers, 0.04 kPa/l.s and 1.08 kPa/l respectively in asthma. Thus, the degree of measurement bias for reactance appeared to increase with worsening airflow obstruction. These differences between IOS and AOS were also reproduced using a phantom 3D printed airway resistance model and with a standard volume reactance. Hence, their data confirmed the comparative bias for AOS in regard to relatively higher AX values even in healthy subjects, albeit with a greater difference in AX occurring in patients with airflow obstruction.

A robust aspect of our data was being able to report pre- and post-bronchodilator values when comparing IOS and AOS. As expected we found that the mean differences between devices were smaller for post- compared to pre-bronchodilator values of AX and Fres. In other words, giving a bronchodilator improves the variance between devices. We also found that percentage reversibility response to salbutamol was relatively larger in asthma compared to COPD for AX but not for FEV_1_. The apparent lack of difference in reversibility between asthma and COPD for FEV_1_ may explained by patients with asthma having a well-preserved pre-bronchodilator FEV_1_ of 88% predicted, such that there was relatively little room for improvement in response to salbutamol. The greater sensitivity of FOT than spirometry in detecting reversibility is well documented and may be explained by the effect of deep inspiration with spirometry which removes the prevailing vagal bronchomotor tone [21]. It has been shown previously in response to either bronchoconstrictor or bronchodilator stimuli that FOT has an excellent signal-to-noise ratio expressed as standardised response means, in both asthma and COPD [22, 23].

A potential limitation of our study is that we did not measure serial changes to see if the degree of bias remains constant over time. Without such longitudinal data, we are unable to speculate whether the measurement bias would remain constant over time. We also acknowledge that we did not have any information on exacerbation history in our patients. However, in patients with asthma, ACQ has been shown to being a strong predictor of future exacerbations [24]. Moreover, AX and R5–R20 are known to be predictive of asthma control and exacerbations [9, 11]. We routinely perform IOS in addition to spirometry in all patients with asthma and COPD who attend our clinic. However, we did not evaluate within breath analysis of impedance as this is more of a research tool and something which is not performed in clinic. Aside from the reactance bias, there are some other key differences between AOS and IOS devices, in particular the Tremoflo being cheaper, portable and more user friendly for operator and patient alike.

In summary, our study showed evidence of better agreement for resistance than reactance when comparing IOS and AOS, perhaps inferring that AOS may be more sensitive at measuring reactance in patients with airflow obstruction.

## Electronic supplementary material

Below is the link to the electronic supplementary material.

Supplementary material 1 Figure E1 Scatter plot in all patients (i.e. asthma and COPD) of post-bronchodilator AX and X5 vs FEV_1_% predicted showing linear regression line of best fit and 95% CI for IOS and AOS shown separately. The R^2^ values for the regression model were A) R^2^=0.51, B) R^2^=0.35, C) R^2^=0.53, D) R^2^=0.36 (p<0.001 for all regression models). The conversion factor from kPa to cmH_2_O is x 10.2. (EPS 7912 kb)
